# Establishment and characterization of a replication-restricted modified African swine fever virus

**DOI:** 10.1128/spectrum.02229-25

**Published:** 2025-12-23

**Authors:** Tomoya Kitamura, Kentaro Masujin, Mitsutaka Ikezawa, Takehiro Kokuho

**Affiliations:** 1 National Institute of Animal Health (NIAH), National Agriculture and Food Research Organization (NARO), Kodaira, Tokyo, Japan; Changchun Veterinary Research Institute, Chinese Academy of Agricultural Sciences, Changchun, China

**Keywords:** African swine fever virus (ASFV), immortalized porcine kidney macrophage (IPKM), replication-restricted virus, S273R gene, vaccine

## Abstract

African swine fever is a fatal, febrile, infectious disease that affects pigs and wild boars and is caused by infection with African swine fever virus (ASFV). The current pandemic strain, belonging to genotype II, first emerged in Georgia in 2007 and subsequently spread across Russia, Europe, Asia, and the Caribbean. Developing live attenuated viruses represents the most promising vaccine strategy; however, biosafety and biosecurity concerns due to long-term persistence of attenuated viruses in immunized animals may lead to secondary transmission or reversion to virulence. To address this concern, we developed a replication-restricted ASFV by deleting the S273R gene that encodes an essential factor for core shell formation and viral maturation within host cells. The resulting strain, AQSΔS273R, was unable to replicate in natural host cells because these cells lack a gene that complements the function of S273R. To enable propagation of infectious progeny *in vitro*, we established S273R gene-complemented immortalized porcine kidney macrophages, which are competent to support virus production. In animal trials, pigs immunized with AQSΔS273R exhibited no clinical signs or viremia. In the challenge study, vaccination with replication-restricted ASFV (AQSΔS273R) conferred approximately 30% protection, with partial reduction of clinical signs compared with controls. These findings demonstrate the feasibility of a novel approach for developing replication-restricted ASFV vaccine candidates with enhanced biological safety.

**IMPORTANCE:**

To date, no reliable African swine fever (ASF) vaccines are available. Although some attenuated African swine fever viruses (ASFVs) have been approved for field applications and have attracted attention as potential vaccine candidates, their long-term persistence in inoculated animals raises concerns about virulence reversion or genetic recombination in field settings. In this study, we developed a novel approach to generate safer vaccines by creating a replication-restricted, S273R gene-deleted virus in combination with genetically modified host cells stably expressing the S273R gene. Not only did this mutant virus fail to induce any clinical signs in immunized pigs, but it also partially protected them against challenge with virulent ASFV. These results demonstrate that this newly developed replication-restricted ASFV strain is expected to be a promising and biologically safe vaccine candidate against ASF.

## INTRODUCTION

African swine fever virus (ASFV) is the causative agent for African swine fever, a febrile and lethal infectious disease affecting pigs and wild boars. ASFV belongs to the nucleocytoplasmic large DNA virus group and is currently the only known member of the genus *Asfivirus* of the family *Asfarviridae*. The virion is 175–215 nm in diameter and consists of a five-layered structure: a nucleoid, a core shell, an inner envelope, a capsid, and an outer envelope, in this order from the inside to the outside (1). The genome in the nucleoid, made up of 190 kbp of double-stranded DNA, encodes over 150 genes (2). ASFVs are classified into at least 23 genotypes on the basis of the genetic diversity of the partial sequence of the B646L gene, which encodes the p72 protein (3, 4).

For a long time, ASFV was endemic to the African continent. However, in 2007, genotype II viruses suddenly appeared in Georgia, then subsequently spread into Russia, Eastern Europe, Asia, and the Caribbean countries (5–8). Note that, following the first report of the outbreak in China in 2018, another distinct outbreak caused by the genotype I strain of ASFV was confirmed in 2021 (9). Even more importantly, further outbreaks of the disease caused by the infection with newly emerging recombinant ASFVs of chimeric genotype (genotype I/II) that show a mosaic pattern of genotype I and II genomes were reported in China (2023), Vietnam, and Russia (2024 [10–12]), causing drastic economic losses to the global pig industry.

Until recently, no ASF vaccine has been commercially available since the disease was first reported in East Africa in 1921 (13). However, several live attenuated vaccines (LAVs) have been officially approved for clinical use in Vietnam recently (14, 15). Although these LAVs seem to protect animals from infection with virulent ASFV, the vaccine viruses persist in the bodies of inoculated animals for a month or more after inoculation. The prolonged viral persistence raises concerns about the potential reversion of virulence, and the instability of the ASFV genomic sequence during replication in host cells may increase the risk of virulence reversion. Recent studies have also shown that some attenuated ASFV strains may revert to virulence after *in vivo* passages, raising further biosafety concerns (16–19). These findings underscore the genetic instability of ASFV and emphasize the need for safer, replication-restricted vaccine approaches.

Replication-restricted viruses, which lack the essential gene(s) required for propagation of infectious progeny in infected cells, have been developed as LAVs for various viral diseases, including influenza A, Japanese encephalitis, COVID-19, and Ebola hemorrhagic fever (20–25). In these studies, replication-restricted viruses successfully induced protective immunity against challenge infections. Importantly, these viruses are considered safer than their parental strains because of their reduced ability to propagate in host cells. However, replication-restricted ASFV has not yet been engineered. Therefore, in this study, we aimed to create an attenuated ASFV that does not efficiently replicate in host animals under field conditions.

In this study, we targeted the S273R gene to attenuate the replication capacity of ASFV. The S273R protease is responsible for processing the structural polyproteins pp220 and pp62, whose cleavage products are essential for proper core shell formation and viral morphogenesis (26–28). Because deletion of S273R severely impairs viral replication while still permitting the formation of non-infectious particles, it represents a promising target for generating replication-restricted ASFV.

## RESULTS

### Generation of the S273R-deficient ASFV

**Fig 1:**
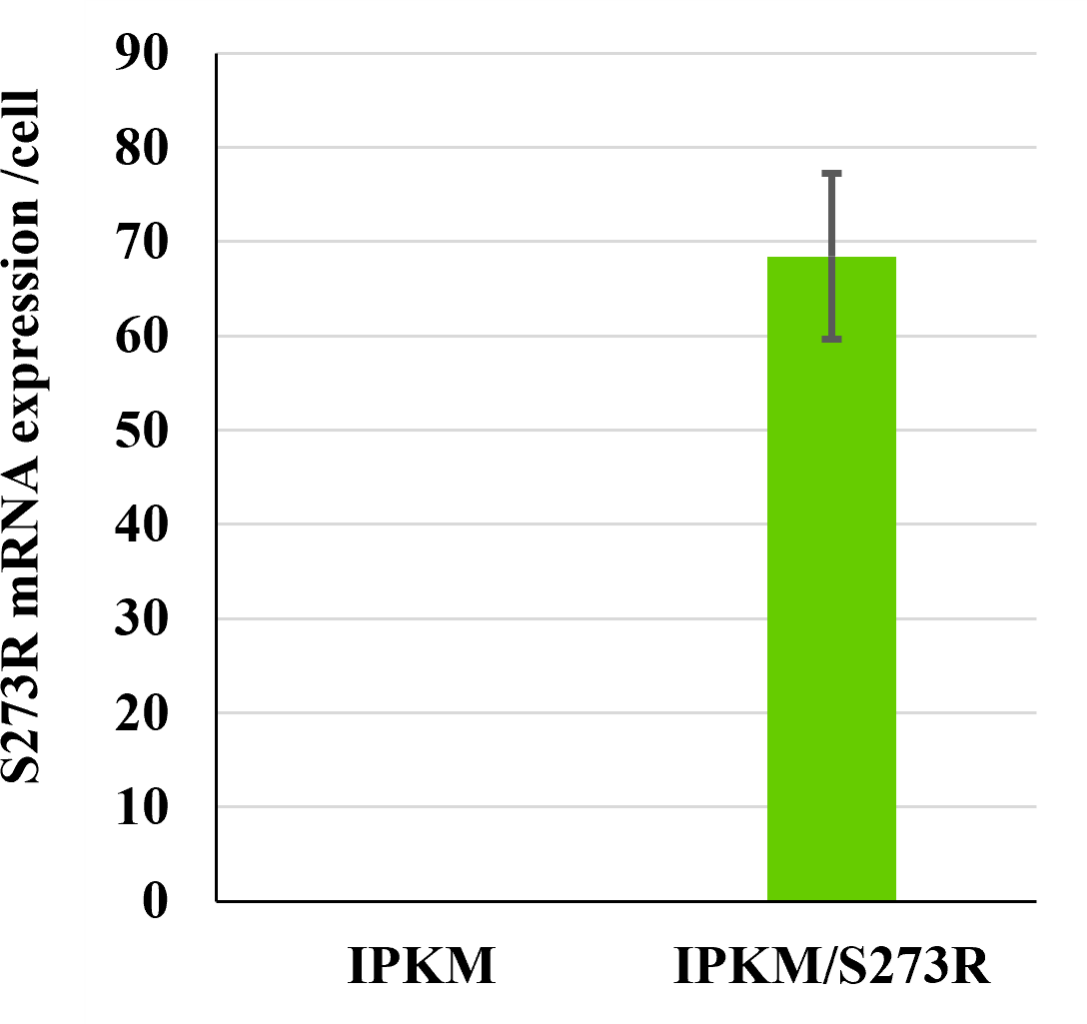
S273R mRNA expression in IPKM/S273R cells. mRNA expression levels of S273R in IPKM and IPKM/S273R cells were measured using real-time reverse transcription PCR. Copy numbers per cell (mean ± standard deviation) from three independent assays were calculated from a standard curve generated using an S273R-inserted plasmid. IPKM, immortalized porcine kidney macrophage.

**Fig 2:**
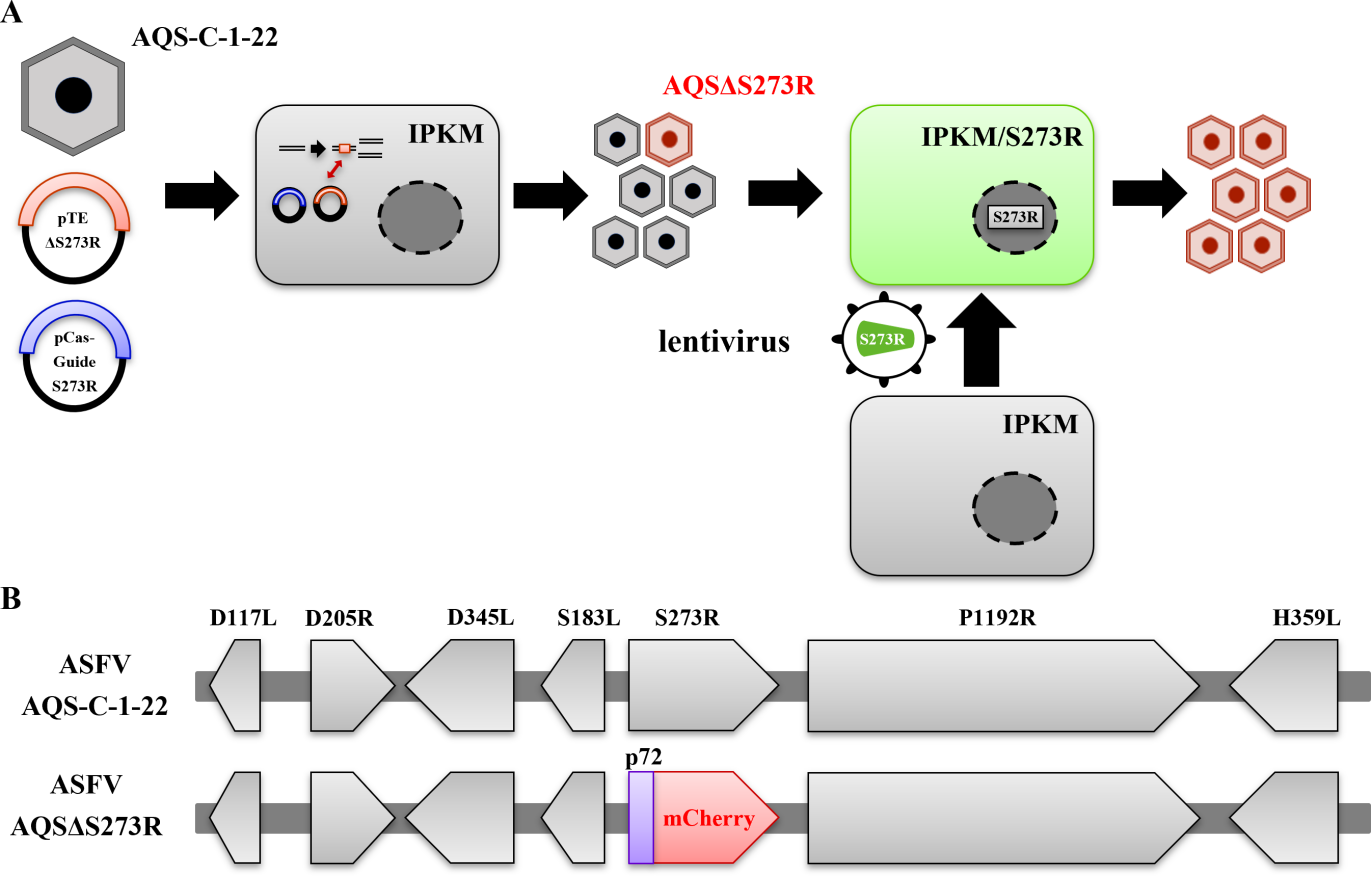
Schematic depicting the generation of replication-restricted ASFV. (**A**) For homologous recombination, AQS-C-1-22 was used to infect IPKM cells that had been transfected with pTEΔS273R and pCas-guide S273R. IPKM cells stably expressing S273R were generated through lentiviral transfection and subsequently used to isolate AQSΔS273R by the limiting dilution method. (**B**) To generate AQSΔS273R, the mCherry gene downstream of the ASFV p72 promoter was inserted into the AQS-C-1-22 genome at the original position of the S273R gene by homologous recombination. ASFV, African swine fever virus.

The S273R gene was selected as a target for designing replication-restricted ASFV because its encoding protein catalyzes two distinct viral structural proteins, pp220 and pp62, that are indispensable for core shell formation during particle maturation (26, 27). To rescue the S273R deletion mutant (AQSΔS273R), we modified the immortalized porcine kidney macrophage (IPKM) cell line and established its transformant stably expressing supplemental S273R gene products. The established IPKM/S273R cell line exhibited a higher level of S273R mRNA expression than its parental cell line (Fig. 1). The method for generating the S273R-deficient ASFV mutant (AQSΔS273R) is illustrated in Fig. 2A. Briefly, AQSΔS273R was created by plasmid-based homologous recombination in IPKM cells. Following isolation, the purity of AQSΔS273R was confirmed by next-generation sequencing. No contamination of the remaining parental virus and unintended nucleotide mutations in the genome sequence was detected.

### Replication of AQSΔS273R

**Fig 3:**
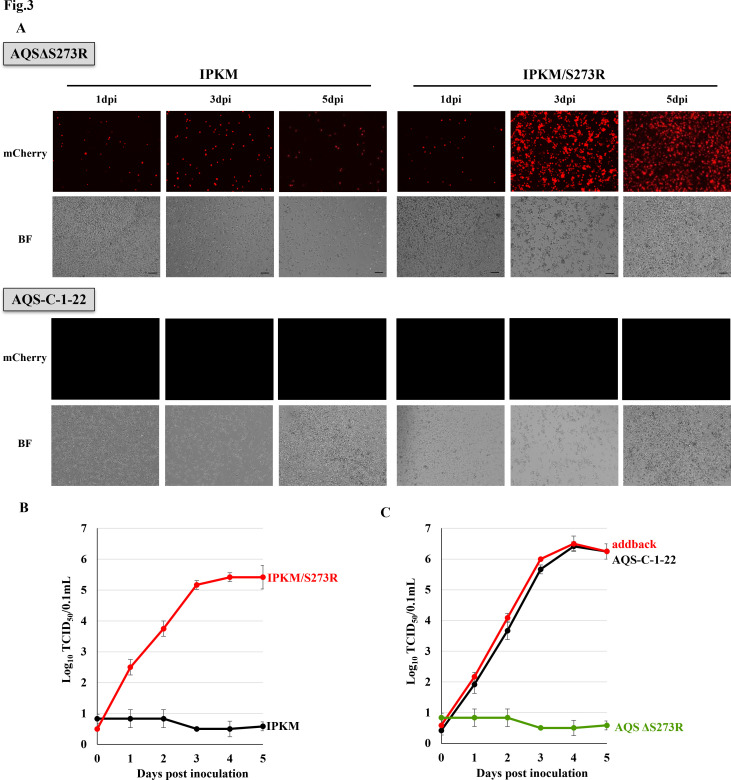
*In vitro* growth of AQSΔS273R, parental AQS-C-1-22, and addback virus. (**A**) IPKM or IPKM/S273R cells were inoculated with AQSΔS273R or AQS-C-1-22 at an MOI of 0.05. These cells were observed under a fluorescence microscope at the indicated time points. Red signals indicate mCherry expression. Scale bar: 50 μm.(**B**) IPKM or IPKM/S273R cells were inoculated with AQSΔS273R at an MOI of 0.01. The supernatants were collected at the indicated time points and titrated using IPKM cells. The data represent virus titers (mean TCID_50_/mL ± SD) from three independent assays. (**C**) IPKM cells were inoculated with AQSΔS273R, parental AQS-C-1-22, or the addback virus at an MOI of 0.01. The supernatants were collected daily and titrated. The data represent virus titers (mean TCID_50_/mL ± SD) from three independent assays. MOI, multiplicity of infection; TCID_50_, median tissue culture infectious dose.

To assess the *in vitro* replication profile of AQSΔS273R, we infected both IPKM and IPKM/S273R cells with the virus at an equal initial multiplicity of infection (MOI). In IPKM/S273R cell cultures, fluorescence signals—indicative of viral replication—increased until 5 dpi, by which time nearly all cells exhibited fluorescence. In contrast, fluorescence-positive cells remained scarce in IPKM cultures throughout the observation period (Fig. 3A). A comparative growth analysis at an MOI of 0.01 further demonstrated that AQSΔS273R replicated efficiently in IPKM/S273R cells but showed minimal propagation in IPKM cells (Fig. 3B). To confirm whether the replication deficiency observed in IPKM cells was directly attributed to the S273R gene deletions, we reintroduced the S273R gene into the AQSΔS273R genome by homologous recombination, replacing the marker gene cassette. The resulting virus, designated the S273R addback virus, was isolated, and its replication profile was evaluated in IPKM cells. The S273R addback virus replicated efficiently in IPKM culture, thereby confirming that the S273R gene is essential for robust viral replication in these host cells (Fig. 3C).

### Morphological analysis of AQSΔS273R particles

**Fig 4:**
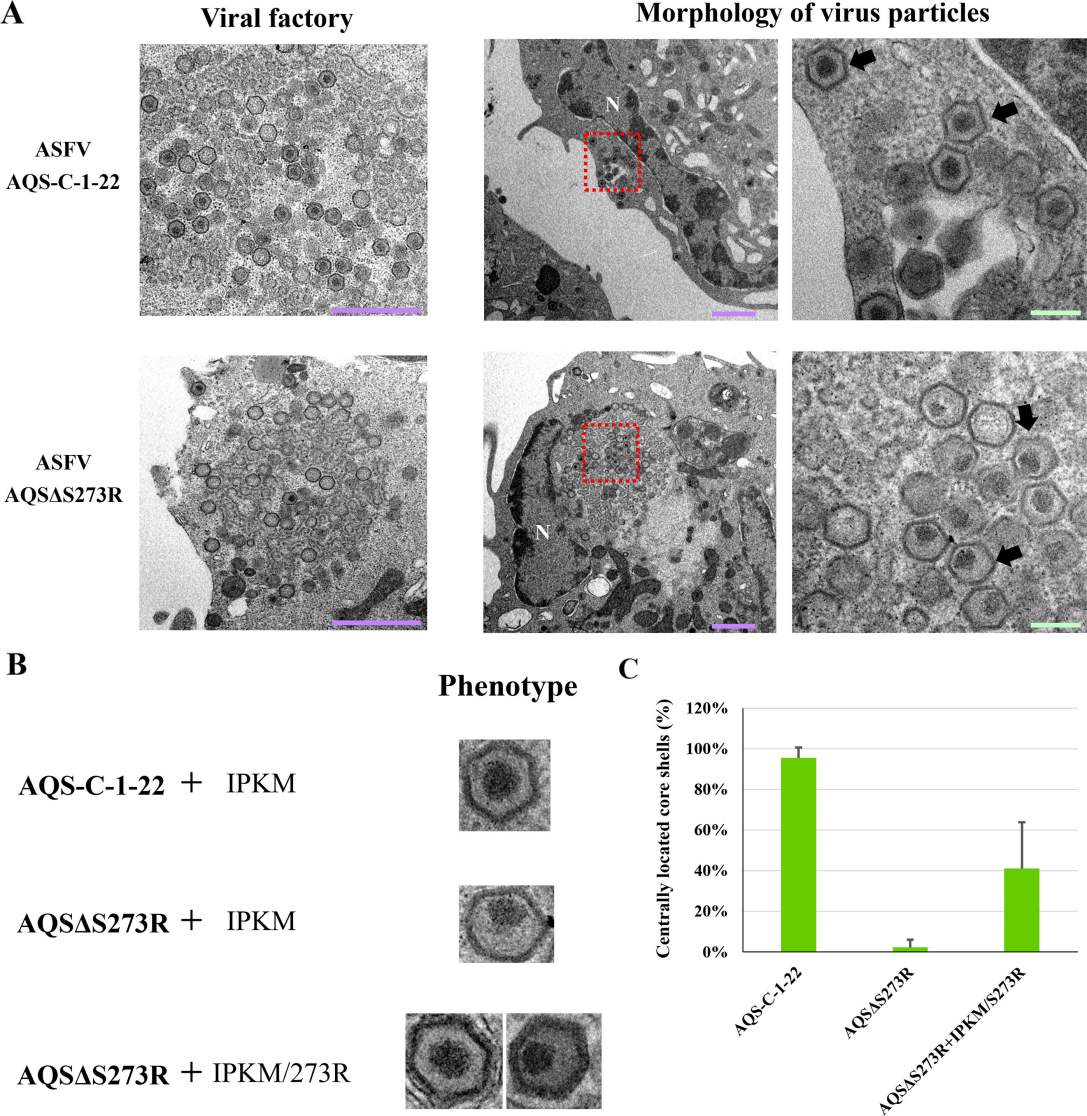
Effect of S273R deficiency on viral particle assembly. (**A**) IPKM cells were infected with AQS-C-1-22 or AQSΔS273R at an MOI of 5 and fixed with paraformaldehyde and glutaraldehyde after 20 h of incubation. Following osmium fixation and dehydration, the cells were embedded in EPON resin. Ultrathin sections were stained with uranyl acetate and lead citrate. The core shells of AQS-C-1-22 virions (upper panel) were centrally located, whereas the core shells of AQSΔS273R virions (lower panel) appeared off-center. Areas highlighted by red rectangles are shown at higher magnification on the right. The arrows indicate viral particles; the purple and green bars represent 1.0 µm and 200 nm, respectively. (**B**) Morphology of AQS-C-1-22 and AQSΔS273R virions assembled in IPKM cells (upper) and IPKM/S273R cells (middle), and AQSΔS273R virions assembled in IPKM/S273R cells (bottom). AQS-C-1-22 virions assembled in IPKM cells displayed the typical icosahedral shape of ASFV with centrally located core shells. In contrast, AQSΔS273 virions exhibited off-center core shells. Notably, AQSΔS273 virions assembled in IPKM/S273R cells displayed both centrally and peripherally positioned core shells. (**C**) Percentage of AQS-C-1-22 and AQSΔS273R virions assembled in IPKM cells and IPKM/S273R cells, and AQSΔS273R virions assembled in IPKM/S273R cells. In each condition, 30 virus particles were counted, and the percentage of those with centrally located cores is shown. The data represent the percentage of centrally located core shells (mean ± SD) from three independent assays.

The deletion of the S273R gene has previously been reported to be associated with the formation of abnormally shaped particles with a decentralized core shell (27). To investigate whether AQSΔS273R exhibits a similar phenotype, we examined AQS-C-1-22 and AQSΔS273R particles in infected IPKM cells using transmission electron microscopy. As expected, AQS-C-1-22 formed particles with a normal morphology, characterized by a centrally located core shell (Fig. 4A). In contrast, the majority of AQSΔS273R particles displayed a distorted core shape displaced from the center. Notably, when AQSΔS273R was assembled in IPKM/S273R cells, the resulting particles exhibited mixed morphologies, including both normal and decentralized core shells (Fig. 4B and C). The proportion of normal core shells was approximately 40%. These findings suggest that the absence of S273R contributes to the development of an abnormal core shell structure, consistent with previous observations.

### Virulence of AQSΔS273R in pigs

**Fig 5:**
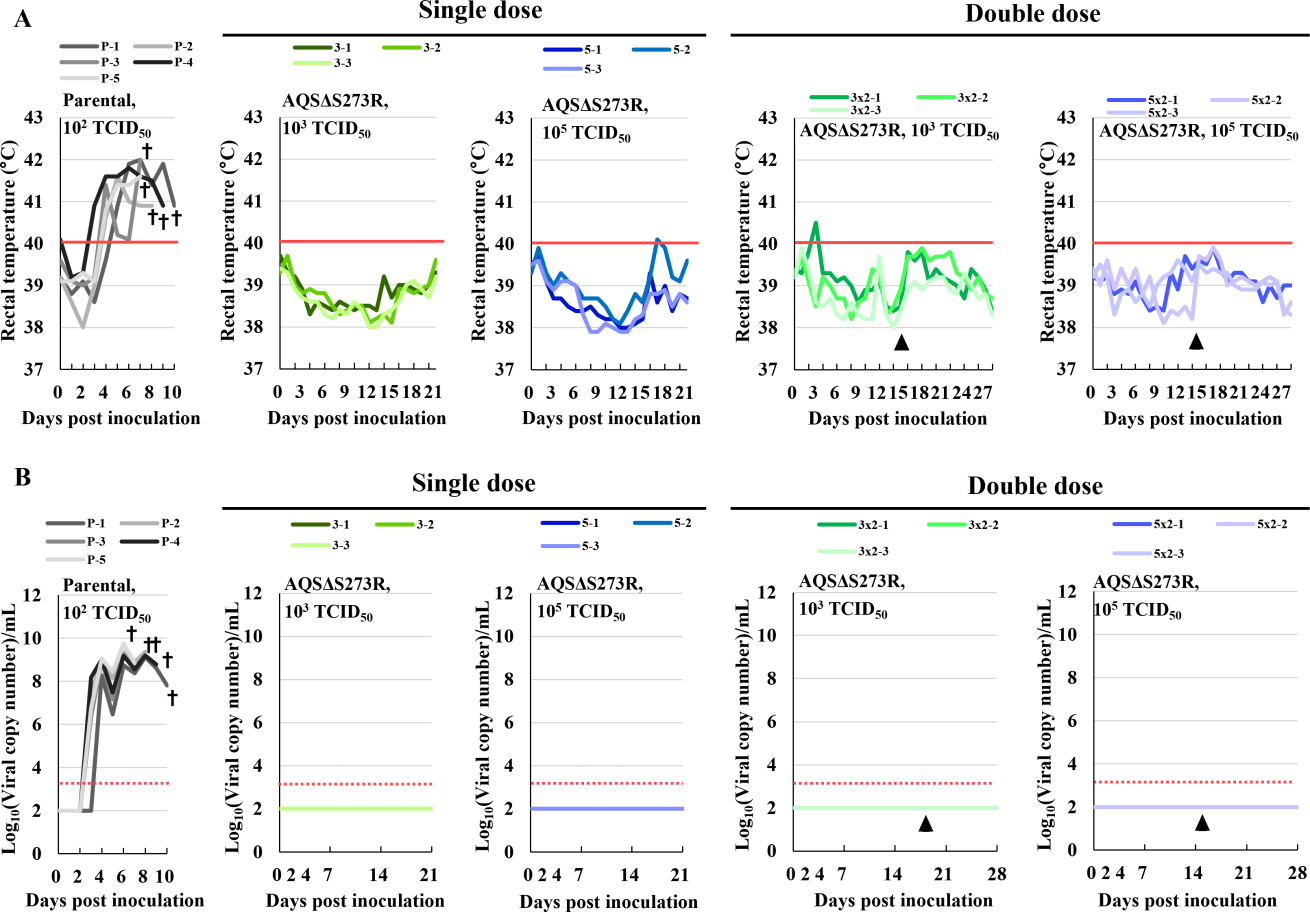
Virulence of AQSΔS273R in pigs. (**A**) Rectal temperatures of pigs inoculated with a single or double dose of AQSΔS273R. The green and blue colors indicate groups treated with a lower dose (10^3^ TCID_50_) and a higher dose (10^5^ TCID_50_) of the virus per injection. Fever was defined as temperatures above 40℃ (red line). The gray-black colors represent the control group injected with 10^2^ TCID_50_ of the parental virulent strain. (**B**) Viral copy numbers in whole blood were measured using quantitative PCR. The dotted lines indicate the detection limit of the assay. Each experimental group is indicated in the same colors. The crosses indicate animals that died or were euthanized; the triangles mark the day of second immunization.

To assess the virulence of AQSΔS273R *in vivo*, pigs were intramuscularly inoculated with the virus at a single dose of 10^3^ or 10^5^ median tissue culture infectious dose (TCID_50_) per animal (*n* = 3 each). None of the inoculated animals presented clinical signs such as fever and viremia, and all the animals survived for 21 dpi (Fig. 5A and B). Additionally, we tested a double-dose regimen, administering 10^3^ or 10^5^ TCID_50_ on both day 0 and day 14 (*n* = 3 each). The results were similar to those obtained after single-dose inoculation, with no clinical signs or mortality.

In contrast, the control group (*n* = 5), inoculated with the parental virulent AQS-C-1-22 at a dose of 10^2^ TCID_50_ per animal, exhibited severe pyrexia (>40°C) by 5 dpi (Fig. 5A) and developed clear clinical signs such as anorexia and diarrhea. All animals in this group died or were euthanized between 7 and 10 dpi, and viremia was detected from 4 dpi or earlier (Fig. 5B).

### Humoral response in AQSΔS273R-inoculated pigs

**Fig 6:**
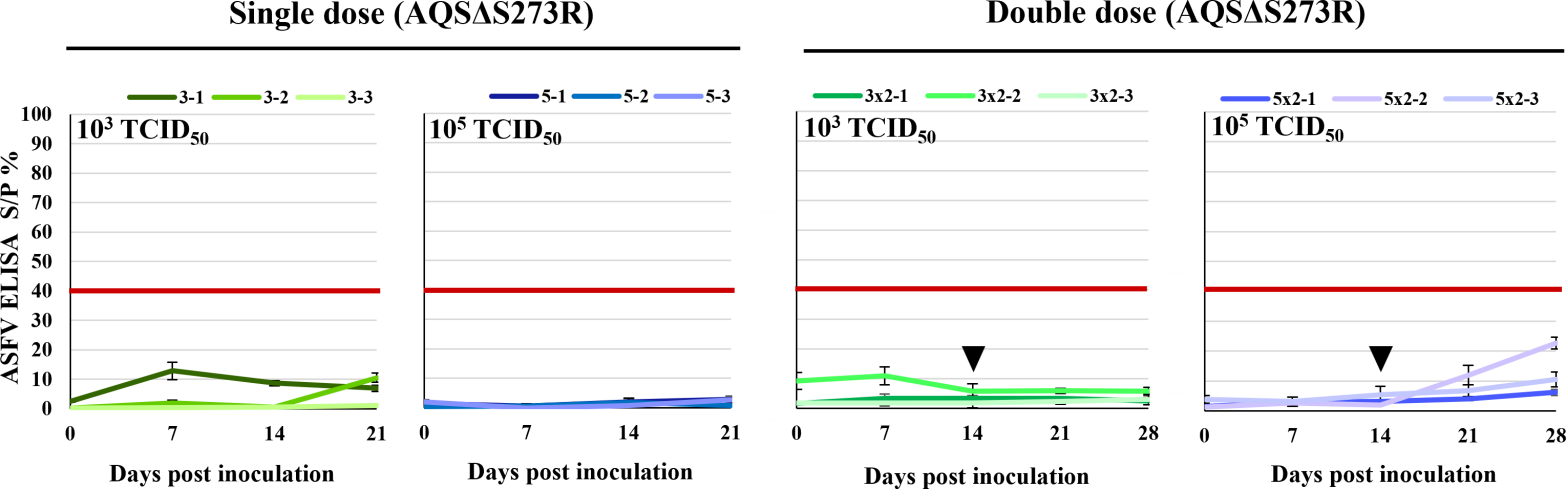
Detection of ASFV-specific antibodies. ASFV-specific serum antibodies in AQSΔS273R-inoculated pigs were detected using an ID Screen African Swine Fever Indirect ELISA kit. The red lines indicate the level deemed “positive” by the assay. Green and blue colors represent the groups treated with 10^3^ and 10^5^ TCID_50_ of AQSΔS273R, respectively. The left and right panels denote the single-dose- and double-dose-treated groups. The black triangles indicate the day of second immunization. The optical density (OD) was measured at 450 nm on a microplate reader. The sample-to-positive (S/P) ratio was calculated using the following equation: (sample OD − negative control OD) / (positive control OD − negative control OD). The data represent the S/P ratio (mean ± SD) from three independent assays. Samples with an S/P ratio of 0.4 or higher were considered positive for ASFV-specific antibodies.

Antibody responses in pigs inoculated with AQSΔS273R were assessed using the ID Screen African Swine Fever Indirect Kit (IDvet, Grabels, France). ASFV-specific antibodies were scarcely detected in any of the pigs, irrespective of the inoculation dose or the number of administrations (Fig. 6).

### Protective efficacy of AQSΔS273R against virulent virus challenge

To assess the protective efficacy of AQSΔS273R, pigs previously inoculated with AQSΔS273R were challenged with the parental virulent strain AQS-C-1-22 at a dose of 10^2^ TCID_50_ per animal. The challenge was administered at 21 dpi for the single-dose group and 28 dpi for the double-dose group. Animals were monitored for 30 days post-challenge (dpc), and the outcomes are summarized in Table 1.

**TABLE 1:** Summary of the animal experiment for assessment of pathogenicity and protection efficacy of AQSΔS273R

Virus	Dose	Pig	Survived or dead^*a*^	Maximum of rectal temperature (°C)			Maximum of viral copy number (10^*n*^/mL)		
				Pre- challenge	Post- challenge	Post-mean	Pre- challenge	Post- challenge	Post-mean
Parental	Single, 10²	P-1	D	39.2	42	41.8	ND^*c*^	9.1	9.3
		P-2	D	39.2	41.6			9.4	
		P-3	D	39.6	42			9.2	
		P-4	D	40.1	41.8			9.2	
		P-5	D	39.1	41.6			9.8	
AQSΔS273R	Single, 10³	3-1	S	39.7	41.1	41.6	ND^*c*^	5.9	7.2 (**P = 0.04)^*b*^
		3-2	D	39.7	41.3			8.3	
		3-3	D	39.4	42.3			7.3	
	Single, 10⁵	5-1	D	39.7	41.3	41.5	ND^*c*^	7.0	7.8 (** P = 0.04)^*b*^
		5-2	D	40.1	41.6			8.4	
		5-3	D	39.6	40.7			8.1	
	Twice, 10³	3x2-1	D	40.5	41.5	41.2	ND^*c*^	7.3	7.0 (**P = 0.04)^*b*^
		3x2-2	D	39.9	41.8			8.3	
		3x2-3	S	39.9	41.2			5.5	
	Twice, 10⁵	5x2-1	S	39.9	40.9	41.0 (**P = 0.03)^*b*^	ND^*c*^	5.7	7.2 (**P = 0.04)^*b*^
		5x2-2	D	39.9	41.1			7.6	
		5x2-3	D	39.6	41.1			8.4	

^*a*^ D; dead, S; survived.

^*b*^ Mann–Whitney *U* test.

^*c*^ ND, not detected.

**Fig 7:**
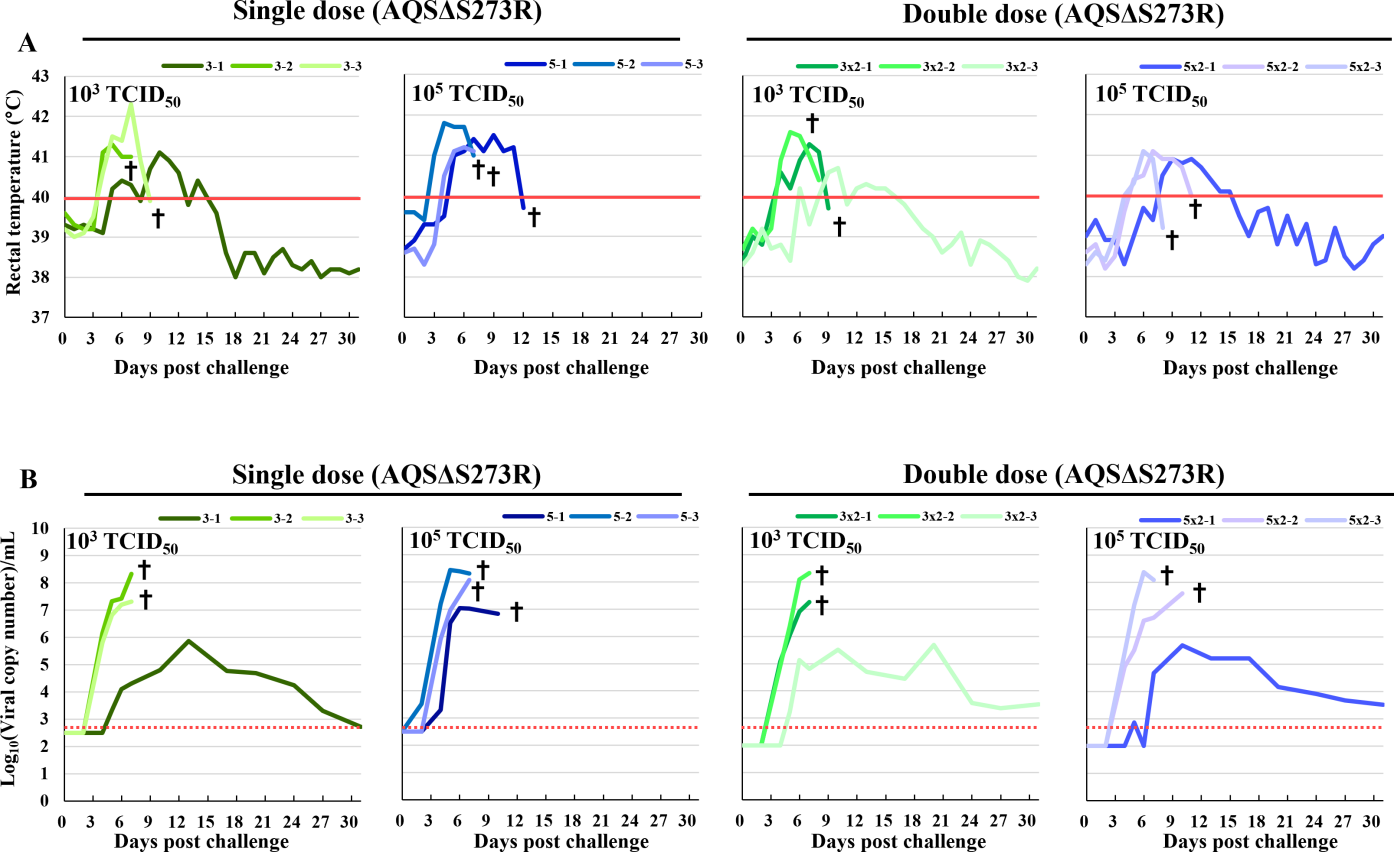
Assessment of the protective efficacy of AQSΔS273R in pigs. (**A**) Rectal temperature after challenge with the parental virus. Fever was defined as a temperature above 40℃ (red line). (**B**) Viral copy numbers in whole blood after challenge with the parental virus, as measured using quantitative PCR. The dotted lines indicate the detection limit of the assay. Green and blue indicate animal groups immunized with either 10^3^ or 10^5^ TCID_50_ of AQSΔS273R, respectively. The number of inoculations is displayed above each graph. All inoculated pigs were challenged with 10^2^ TCID_50_ of the parental virulent strain AQS-C-1-22 at 21 dpi for the single-dose groups (left panels) and 28 dpi for the double-dose groups (right panels). Crosses indicate dead or euthanized pigs.

Only three out of 12 pigs survived the challenge: one that received a single dose of 10^3^ TCID_50_ of the AQSΔS273R, one that received a double dose of 10^3^ TCID_50_ of the AQSΔS273R, and one that received a double dose of 10^5^ TCID_50_ of the AQSΔS273R. All other pigs succumbed by 12 dpc. While all pigs exhibited high fever (>40°C) and viremia following challenge, these clinical signs were transient in the surviving pigs and resolved by 15 dpc (Fig. 7). ASFV-specific antibodies were detected only in the sera of surviving pigs at 2 weeks post-challenge (data not shown). Notably, fever and viremia were less severe in AQSΔS273R-inoculated pigs compared to naive controls (Fig. 7; Table 1).

Gross pathological lesions, including splenomegaly and severe hemorrhage in the gastrohepatic lymph nodes, were observed in all euthanized and deceased pigs but were absent in the survivors.

## DISCUSSION

Recent studies have developed several attenuated ASFV strains as promising live vaccine candidates, demonstrating high efficacy against infections with virulent field strains (14, 15, 29–31). However, concerns remain regarding biosafety, as some of these vaccine candidate strains have been shown to persist in inoculated animals for unexpectedly long periods. This persistence raises the risk of reversion to virulence through spontaneous molecular events. In this study, we propose a novel approach for generating safer vaccine candidates by deleting essential viral genes to create replication-restricted, genetically modified ASFVs. Ideally, such viruses would be rapidly cleared from immunized hosts while still eliciting robust immunity. To this end, we constructed a replication-deficient ASFV mutant lacking the S273R gene using genetically engineered IPKM cells that stably expressed the deleted gene product, thereby allowing virus replication.

The S273R gene encodes a cysteine proteinase that is essential for processing the viral structural proteins pp220 and pp62 during viral core shell formation (26, 27). Deletion of this gene disrupts virus maturation and replication. In line with previous findings, the resulting AQSΔS273R mutant lacked replication and propagation activities in wild-type IPKM cells (Fig. 3). Moreover, pigs inoculated with 10^5^ TCID_50_ of AQSΔS273R showed no clinical signs such as pyrexia and viremia (Fig. 5), indicating that viral replication was severely suppressed both *in vitro* and *in vivo*.

Following the challenge with the parental virulent ASFV strain, pigs immunized with AQSΔS273R exhibited lower peak body temperatures and reduced viremia compared to unvaccinated controls (Fig. 7; Table 1), indicating partial protective immunity. Although ASFV-specific antibody titers did not significantly increase (Fig. 6), mRNA expression of p30 and p72 (data not shown) suggests that these proteins, along with pp62, were likely synthesized, potentially contributing to immunity despite limited antibody responses. This suggests that protection is likely mediated by cellular immune responses rather than humoral responses, consistent with recent studies (32, 33). Although our results suggest a role for cellular immunity, direct evidence is still lacking; therefore, future work should evaluate these responses by evaluating ASFV-specific CD8^+^ T-cell proliferation and IFN-γ production to confirm the proposed mechanism of protection. However, due to the lack of direct evidence supporting our interpretation, further elucidation of the protective mechanism mediated by ASFV-specific cellular immunity is necessary.

Our findings reveal that AQSΔS273R exhibits suboptimal immunogenicity, necessitating further efforts to improve its efficacy. This limited efficacy is likely to result from insufficient presentation of viral antigens by antigen-presenting cells (APCs), which impair adaptive immune responses. Dose escalation (e.g., 10⁷ TCID₅₀) and multi-dose prime–boost regimens could be promising strategies to elicit stronger immune responses. Previous studies on inactivated ASFV vaccines have suggested that, while not fully protective due to lacking MHC class I-dependent immune activation, improved antigen-specific responses (32). Co-administration with adjuvants may also enhance the efficacy of AQSΔS273R, as suggested in prior studies (33).

Live attenuated vaccines with deletions of non-essential gene(s) can induce robust immunity through repeated rounds of replication in the host. However, these vaccines inherently carry risks of unintended biological changes in transmissibility and pathogenicity due to accumulation of spontaneous mutations during *in vivo* passage. Recent reports have suggested such alterations with currently used vaccine strains (16, 17). Replication-restricted viruses are not entirely risk-free, as mutations may still arise during their limited replication in the host. However, because the number of replication cycles *in vivo* is inherently limited, mutation rates are substantially reduced, conferring a high degree of safety of these viruses when used as vaccines in practical applications. Additionally, unlike viral-vectored or subunit vaccines, replication-restricted virus vaccines express a comprehensive set of ASFV antigenic epitopes, which are processed and presented by APCs in infected hosts. This process may enable broader immune responses, potentially critical for achieving full protection against ASFV while ensuring superior safety compared to conventional live attenuated vaccines.

In conclusion, we have established a novel platform for the generation of replication-restricted ASFVs by utilizing genetically modified IPKM cells that complement essential viral gene deletions. This system enables the efficient development of ASFV mutants with replication-restricted phenotypes, offering valuable tools not only for creating safe vaccines but also for advancing our understanding of ASFV pathogenesis.

## MATERIALS AND METHODS

### Cells

IPKM cells were established in our institute by immortalizing porcine primary cultures of kidney macrophages with recombinant lentivirus vectors expressing either SV40 large T antigen or porcine telomerase reverse transcriptase, as previously described (34). IPKM cells are highly susceptible to field ASFV isolates and cell-adapted ASFV isolates (35–37). These were routinely maintained in Dulbecco’s modified Eagle’s medium (DMEM, 08459-35; Nakalai Tesque, Kyoto, Japan) supplemented with 10% fetal bovine serum (10270-106; Gibco, Waltham, MA, USA), 10 μg/mL bovine insulin (I0516; Merck, Darmstadt, Germany), 25 μM monothioglycerol (195-15791; Wako, Osaka, Japan), and antibiotics (168-23191, Wako). The cells were grown in cell culture plates and flasks for suspension culture (Sumitomo Bakelite, Tokyo, Japan). Lenti-X 293T cells (632180; TaKaRa Bio, Shiga, Japan) were maintained in DMEM supplemented with 10% fetal bovine serum.

### Virus

The virulent ASFV strain AQS-C-1-22 (genotype II) was isolated from an illegally imported pork product seized by the animal quarantine service of Japan (36, 38). AQS-C-1-22 was propagated in IPKM cells and used for experiments. All experiments involving ASFV strains were performed in the biosafety level 3 facility of the institute accredited by the national authority of Japan.

### Establishment of S273R-expressing IPKM cells

The S273R gene of AQS-C-1-22 was amplified using specific primers and cloned into the pLVSIN-CMV Pur lentiviral vector (6183, TaKaRa Bio) between the EcoRI and BamHI digestion sites (pLVSIN-S273R). To generate recombinant lentivirus, pLVSIN-S273R and Lentiviral High Titer Packaging Mix (6194, TaKaRa Bio) were co-transfected into Lenti-X 293T cells. The S273R-expressing lentivirus was inoculated into IPKM cells, and the expressing cells were selected using puromycin selection (2 μg/mL, ant-pr-1; Invivogen, San Diego, CA, USA). The mRNA expression level of S273R was measured using real-time reverse transcription PCR. Briefly, total RNA was extracted from 2 × 10^5^ cells by the CellAmp Direct RNA Prep Kit for RT-PCR (Real Time) (3732, TaKaRa Bio) according to the manufacturer’s protocol. The expression level of S273R was quantified using the One Step TB Green PrimeScript RT-PCR Kit II (RR086A, TaKaRa Bio) with specific primer sets to detect S273R. pLVSIN-S273R was used as a standard in the concentration range of 1.0 × 10^8^–1.0 × 10^1^ copies/µL.

### Generation of AQSΔS273R and S273R addback virus

AQSΔS273R was generated through plasmid-based homologous recombination in IPKM cells. The upstream region of the S273R gene (approximately 1,000 bp), combined with the p72 gene promoter sequence, the mCherry gene, and the downstream region of the S273R gene (approximately 1,000 bp) in this order, was cloned into the pGEM-T Easy vector (pTE-ΔS273R). We also constructed a vector using pCas-Guide (GE100002; ORiGENE, Rockville, MD, USA) as the backbone encoding the Cas9 nuclease gene and S273R-specific guide RNA (pCas-Guide S273R) (39). After transducing pTE-ΔS273R and pCas-Guide S273R into IPKM cells, the cells were infected with AQS-C-1-22 at an MOI of 0.1. At 5 dpi, the viral supernatant was collected, and limiting dilution was performed in IPKM/S273R cells to isolate AQSΔS273R. Viral DNA was extracted from isolated clones with mCherry fluorescence. The gene replacement of the S273R gene with the mCherry gene was confirmed by PCR using the primer sets described in Fig. S1. To reconstitute the S273R gene in AQSΔS273R, a pGEM-T Easy vector containing the entire S273R gene was introduced into IPKM cells, followed by the inoculation of AQSΔS273R. The addback virus was isolated by the limiting dilution method as described above. The primer sets used for the generation of AQSΔS273R and its addback mutant are described in Fig. S1 and Table 1.

### Growth kinetics

AQS-C-1-22, AQSΔS273R, or addback viruses were inoculated into IPKM or IPKM/S273R cells at an MOI of 0.01 and incubated for 60 min at 37°C before being completely removed. The cells were washed and maintained in growth medium. Culture supernatants were collected daily up to 5 dpi. Finally, the TCID_50_ of all the supernatants was determined using IPKM cells as the host cells.

### Next-generation sequencing of the ASFV genome

Genome sequencing was performed as previously described with slight modifications (35–37). Briefly, the collected viral supernatant was centrifuged at 180,000 × *g* at 4°C for 3 h. The resultant pellets were resuspended in 100 µL of phosphate-buffered saline and treated with 250 U of benzonase nuclease (E8263, Merck) at 37°C for 1 h. Viral DNA was extracted using the High Pure Viral Nucleic Acid Kit (11858874001; Roche, Basel, Switzerland) and subjected to next-generation sequencing using the iSEQ 100 Sequencing System (Illumina, San Diego, CA, USA), according to the manufacturer’s protocols. The viral reads were trimmed using the Trimmomatic tool, v.0.36.3 (40), and mapped to the genome of the AQS-C-1-22 isolate (GenBank accession no. LC659087) using the Bowtie2 tool (41). The data processing and analysis were performed using the Galaxy web platform, v.2.3.0 (42). The accession number for AQSΔS273R is LC896302.

### Quantitative PCR

Viral DNA was extracted from whole blood or tissue homogenate using the High Pure Viral Nucleic Acid Kit (Roche). PCR was performed as previously described (43).

### Animal experiments

All pigs received weaner/grower feed and had access to water *ad libitum* throughout the study. The animals were observed daily for clinical signs and/or welfare impairment. All efforts were made to minimize animal suffering and to reduce the number of animals used.

The virulence of AQSΔS273R was assessed using 8-week-old castrated crossbred Landrace × Large White × Duroc male and female pigs, 17 in total, obtained from a commercial health-status herd. All pigs were checked for the absence of ASFV and anti-ASFV antibodies using quantitative PCR (43) and the ID Screen African Swine Fever Indirect Kit (ASFS-2P, IDvet), respectively. The pigs were randomly divided into five groups: four groups of three pigs each and one group of five pigs. The group with five pigs was inoculated intramuscularly with the AQS-C-1-22 strain at a single dose of 10^2^ TCID_50_. The other four groups were inoculated intramuscularly with AQSΔS273R at either single or double doses of 10^3^ or 10^5^ TCID_50_. In the double-dose groups, the second inoculation was performed 14 days after the initial inoculation. The pigs’ clinical signs and body temperature were monitored daily up to 21 dpi for the single-dose groups and 28 dpi for the double-dose groups.

After the virulence experiment, the animals inoculated with AQSΔS273R were used for the challenge experiment. The inoculated animals were challenged by intramuscular inoculation with 10^2^ TCID_50_ of the virulent parental strain AQS-C-1-22, and their clinical signs and body temperature were monitored daily up to 30 dpc.

When pigs markedly reduced their activity and lay down, a humane endpoint was deemed to have been reached, and euthanasia was justified on welfare grounds. Spleens, kidneys, lungs, and gastrohepatic lymph nodes were harvested from dead or euthanized pigs and homogenized in phosphate-buffered saline using a Micro Smash homogenizer (TOMY, Tokyo, Japan).

### Detection of ASFV-specific antibodies

ASFV-specific antibodies in the sera of inoculated or challenged pigs were detected at 0, 7, 14, 21, and 28 dpi and 7, 13, 20, and 28 dpc using the ID Screen African Swine Fever Indirect Kit (IDvet), according to the manufacturer’s protocol. The optical density (OD) was measured at 450 nm on a Nivo microplate reader (PerkinElmer, Waltham, MA, USA). The sample-to-positive (S/P) ratio was calculated using the following equation: (sample OD − negative control OD) / (positive control OD − negative control OD). Samples with an S/P ratio of 0.4 or higher were considered positive for ASFV-specific antibodies.

### Statistics

The viral growth kinetics, maximum of rectal temperature in pigs, and maximum of viral copy number in pigs were analyzed using the Mann–Whitney *U* test to assess statistical significance. Statistical analysis of core shell localization was performed using the Steel–Dwass test to determine the statistical significance of differences.

## Data Availability

The genome sequence of AQSΔS273R (accession no. LC896302) has been deposited in DDBJ/EMBL/GenBank.

## SUPPLEMENTAL MATERIAL

Figure S1 spectrum.02229-25-s0001.tif Schematic representation of the plasmid construction for generating AQSΔS273R or addback viruses.

Table S1 spectrum.02229-25-s0002.docx Primer sets used for AQSΔS273R and addback generation.

ASM does not own the copyrights to Supplemental Material that may be linked to, or accessed through, an article. The authors have granted ASM a non-exclusive, world-wide license to publish the Supplemental Material files. Please contact the corresponding author directly for reuse.
