# Supplementary figures and images for: Establishment and characterization of a replication-restricted modified African swine fever virus
Source: Microbiol Spectr. 2025 Dec 23;14(2):e02229-25. doi: 10.1128/spectrum.02229-25 (PMC12889021; doi:10.1128/spectrum.02229-25)

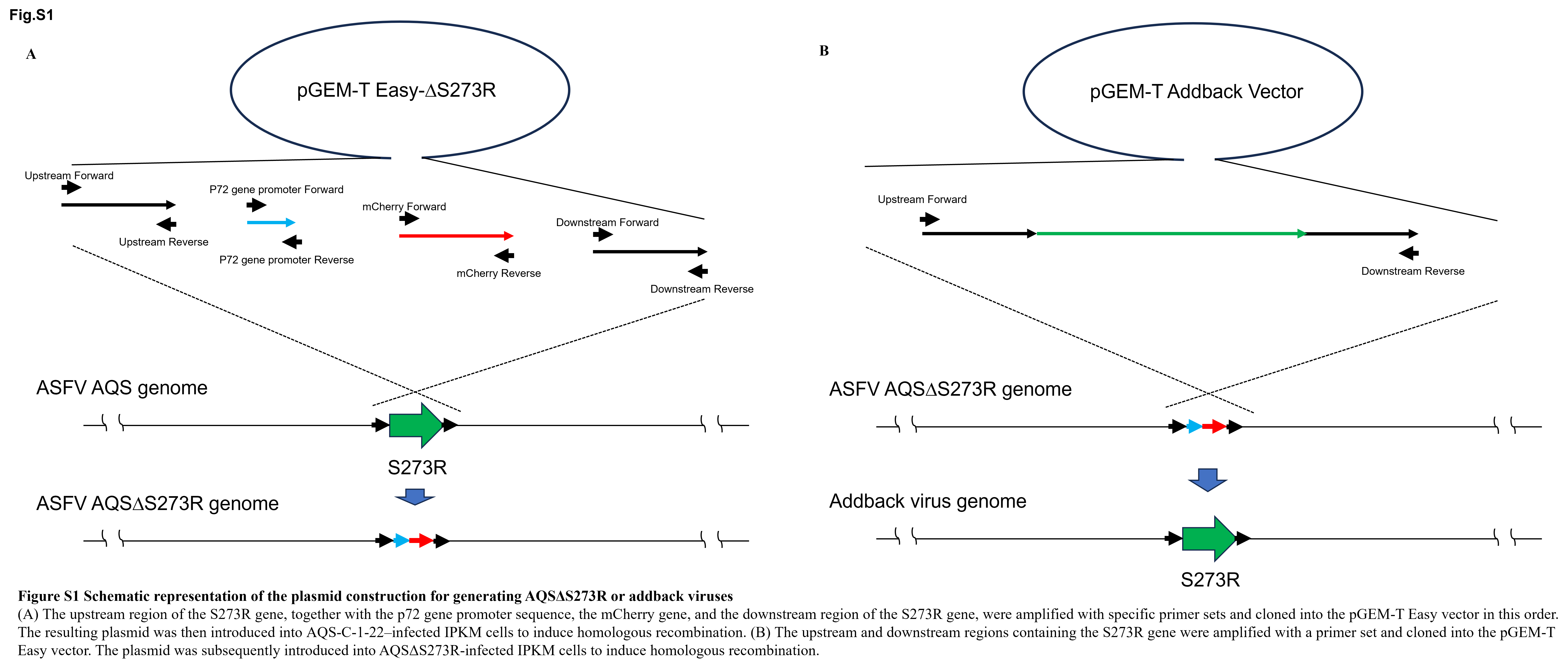

Supplement: Figure S1 — Schematic representation of the plasmid construction for generating AQSΔS273R or addback viruses. [file spectrum.02229-25-s0001.tif]
