# Supplementary material for: Establishment and characterization of a replication-restricted modified African swine fever virus
Source: Microbiol Spectr. 2025 Dec 23;14(2):e02229-25. doi: 10.1128/spectrum.02229-25 (PMC12889021; doi:10.1128/spectrum.02229-25)
Supplement: Table S1 — Primer sets used for AQSΔS273R and addback generation. [file spectrum.02229-25-s0002.docx]

| **Supplementary Table1 List of primer sets used for AQSΔS273R and addback generation** | | |
| --- | --- | --- |
|  |  |  |
| **AQSΔS273R** |  | **Sequences(5'-3')** |
| Upstream | Forward | TAATACGACTCACTATAGGGTAGGTGACAAAACCCATCGG |
|  | Reverse | GACTTTTCCTCCGGCGACCCTTTAGCGGCCACTATACATA |
| Downstream | Forward | TGGACGAGCTGTACAAGTGAACTAATAAAGTTTGAATTC |
|  | Reverse | ATTTAGGTGACACTATAGAAGATAACGGACACGTGTTCA |
| p72 gene promoter | Forward | GGGTCGCCGGAGGAAAAGTC |
|  | Reverse | ATATAATGTTATAAAAATAATTTATTG |
| mCherry | Forward | TTATTTTTATAACATTATATATGGTGAGCAAGGGCGAGGA |
|  | Reverse | TCACTTGTACAGCTCGTCCA |
|  |  |  |
| **Addback** |  | **Sequences(5'-3')** |
| Upstream to Downstream | Forward | TAATACGACTCACTATAGGGTAGGTGACAAAACCCATCGG |
|  | Reverse | ATTTAGGTGACACTATAGAAGATAACGGACACGTGTTCA |
|  |  |  |
| **Confirmation PCR primers** |  | **Sequences(5'-3')** |
| Common | Forward | TTTCCCAGGACTGTTGAGTG |
| S273R check | Reverse | TTTGTAAGATGCTCTGCGCA |
| mCherry check | Reverse | TCACTTGTACAGCTCGTCCA |
